# Life Is Worth Living

**Published:** 1884-05

**Authors:** 


### LIFE *IS* WORTH LIVING.

"DO You think Life Worth Living?" was asked of Mignet, the late French historian, five or six years ago, when he was more than eighty. "I was," he replied, "not born to fortune and have never been rich. Yet if I had the option of taking a fresh start in life on the conditions under which I set out, I should not hesitate to accept the offer. I feel like a person who has witnessed a great drama which is drawing to its close, and who has done his best to understand it. I have not had a box ticket of my own, but I was able to enter the best boxes, which between the acts, is an advantage. It greatly depends upon ourselves whether we go through it in a manner to be satisfied with it or otherwise. Life to me has been a perpetual fête. My greatest trouble has been in seeing friends drop into the gr ve. But I have learned to accept death *en philosophe*."

